# MEASURING A GOOD DEATH: AN ASSESSMENT ACROSS FOUR COUNTRIES

**DOI:** 10.1093/geroni/igac059.2134

**Published:** 2022-12-20

**Authors:** Sarah Clem

**Affiliations:** University of Maryland, Baltimore, Baltimore, Maryland, United States

## Abstract

A good death is often viewed as a common goal in end-of-life care, yet our understanding of what qualifies as a good death is still under development. Previous studies have attempted to conceptualize our understanding of a good death and have highlighted the need to improve our measurement of this construct. The present study examines a measure of a good death across four countries. Data for this study were derived from the Four-Country Survey on Aging and End-of-Life Medical Care (2017), in which participants were sampled using a random digit dial method. The study relies on a sample of 4,239 participants from the US, Italy, Japan, and Brazil. Confirmatory Factor Analysis (CFA) was employed to fit a measurement model to the full sample for a measure of the importance of a good death. Once model fit was deemed sufficient, a multi-group CFA was used to assess invariance across the four countries. Model fit was adequate at the configural and metric levels, highlighting that across the four countries importance of a good death was measured and understood the same. However, the model failed at the scalar level of invariance, implying that indicators for the importance of a good death are valued differently across the four countries. Further developing our understanding of a good death and how it is measured is essential for improving quality end-of-life care. This study identifies a quality measure of good death while simultaneously highlighting the limitations in using such a measure in a transnational context.

